# Performance Analysis in Ski Jumping with a Differential Global Navigation Satellite System and Video-Based Pose Estimation

**DOI:** 10.3390/s21165318

**Published:** 2021-08-06

**Authors:** Ola Elfmark, Gertjan Ettema, Daniel Groos, Espen A. F. Ihlen, Rune Velta, Per Haugen, Steinar Braaten, Matthias Gilgien

**Affiliations:** 1 Department of Civil and Environmental Engineering, Norwegian University of Science and Technology, 7491 Trondheim, Norway; 2 Norwegian Olympic and Paralympic Committee and Confederation of Sports, 5000 Oslo, Norway; steinar.braten@ntnu.no; 3 Department of Neuromedicine and Movement Science, Norwegian University of Science and Technology, 7491 Trondheim, Norway; gertjan.ettema@ntnu.no (G.E.); daniel.groos@ntnu.no (D.G.); espen.ihlen@ntnu.no (E.A.F.I.); 4 Department of Physical Performance, Norwegian School of Sport Sciences, 4014 Oslo, Norway; runevelta@gmail.com (R.V.); perh@nih.no (P.H.); matthiasg@nih.no (M.G.); 5 Center of Alpine Sports Biomechanics, Engadin Health and Innovation Foundation, 7503 Samedan, Switzerland

**Keywords:** ski jumping, sensor fusion, dGNSS, pose estimation, machine learning

## Abstract

This study investigated the explanatory power of a sensor fusion of two complementary methods to explain performance and its underlying mechanisms in ski jumping. A differential Global Navigation Satellite System (dGNSS) and a markerless video-based pose estimation system (PosEst) were used to measure the kinematics and kinetics from the start of the in-run to the landing. The study had two aims; firstly, the agreement between the two methods was assessed using 16 jumps by athletes of national level from 5 m before the take-off to 20 m after, where the methods had spatial overlap. The comparison revealed a good agreement from 5 m after the take-off, within the uncertainty of the dGNSS (±0.05 m). The second part of the study served as a proof of concept of the sensor fusion application, by showcasing the type of performance analysis the systems allows. Two ski jumps by the same ski jumper, with comparable external conditions, were chosen for the case study. The dGNSS was used to analyse the in-run and flight phase, while the PosEst system was used to analyse the take-off and the early flight phase. The proof-of-concept study showed that the methods are suitable to track the kinematic and kinetic characteristics that determine performance in ski jumping and their usability in both research and practice.

## 1. Introduction

**Figure 1:**
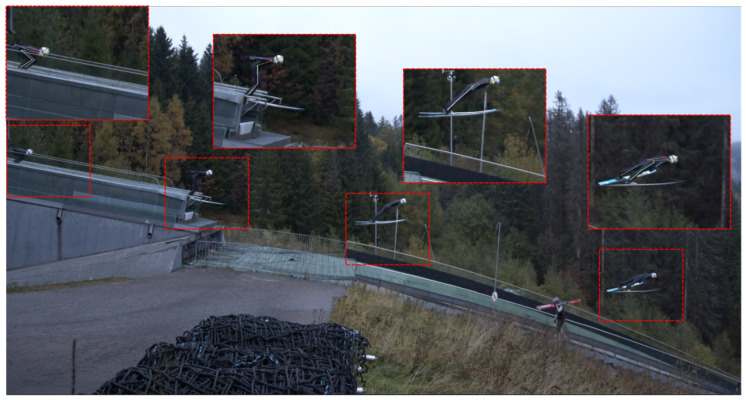
Camera view for the experiment together with examples of the cropped and tracked windows during the in-run, take-off and flight phase.

Ski jumping performance has been a popular research topic over the last twenty years, with some studies dating back as far as 1926 [1]. Performance in ski jumping is quantified in terms of distance from take-off, adjusted for style, start gate and wind conditions. The jump distance is influenced by a variety of factors, such as in-run speed, vertical speed produced in the take-off, wind conditions and the aerodynamics during the flight phase [2]. The current literature considers the take-off to be the most important phase [1,2,3]. A ski jump is commonly analysed by relating the performance outcome to in-run speed (measured with photocells before take-off) together with a qualitative assessment of performance-determining factors during take-off and the early flight phase, using video analysis. Video is typically captured by coaches and the analysis is of a qualitative nature [4]. Typical areas of interest can be seen in Figure 1. As in every sport, the coaching process can be highly subjective [5] and demanding, as the take-off time is approximately 0.3 s, with speeds exceeding 25 m s^−1^ [6,7]. To avoid the pitfalls of qualitative analysis, the objective quantification of sport performance is of great interest [8]. To study sport performance with high internal and external validity, first, the sport should be assessed in competition or in competition-like situations, in the absence of instrumentation that can hamper performance. Second, the performance level of athletes should represent the population to be studied and the number of subjects should be sufficiently high to allow for generalisation of the findings to the given population. Third, the measurement equipment needs to be sufficiently accurate and precise despite the outdoor conditions [9].

Establishing valid measurements of the aspects that impact performance in ski jumping from the start of the in-run to the landing is challenging, as athletes move through a large space, at high speed, in outdoor conditions (variable weather conditions and surroundings). Therefore, many scientific studies have focused on the take-off phase [1,2,3]. For that purpose force plates were mounted under the in-run track to measure the take-off force and timing [10,11,12], and pressure insoles were used to measure force both at take-off and landing [13,14,15]. A force plate has the advantage that it can be used without interfering with the athlete. It can therefore be used in competition, but the data are limited to only the last part of the in-run and the take-off (∼ 10 m) [1]. Pressure insoles, on the other hand, cannot be used in competition but allow the extraction of data from the complete in-run and take-off phase, including parameters such as flight time and data from the landing phase [13]. To capture the effect of the work against the take-off platform on the initial flight phase, measurements of forces were combined with measurement of the athletes’ kinematics during the early flight phase. This combination method allows correlation of the effect of the take-off process with the jump length [10,16]. To quantify the athlete’s motion during the early flight phase in a global metric frame, video-based photogrammetry is applied [2,17,18,19,20,21,22]. Using video analysis, one can extract precise data from a competition without influencing the athletes. As mentioned, cameras are already widely used in ski jumping and no additional hardware is needed [4]. Two dimensional reconstruction of the motion is often sufficient as the general movement is conducted in the sagittal plane [23]. The post-processing of video analysis is usually extremely time consuming, because for the calibration and reconstruction of the athlete’s motion, manual annotation in the pictures is needed [4]. The analysis is also restricted to the area the camera covers, usually take-off and/or the early flight phase. A promising feature of this method is the use of machine learning to train a markerless video-based pose estimation (PosEst) system to recognise a ski jumpers movement and replace the process of manual annotation. This type of technology has evolved considerably in recent years and will reduce the post-processing time substantially [23]. Hence, this method may help to increase the sample size and generalizability of such studies [9]. To date, this type of technology has only rarely been used in ski jumping [24] and other winter sports such as alpine skiing [25].

**Figure 2:**
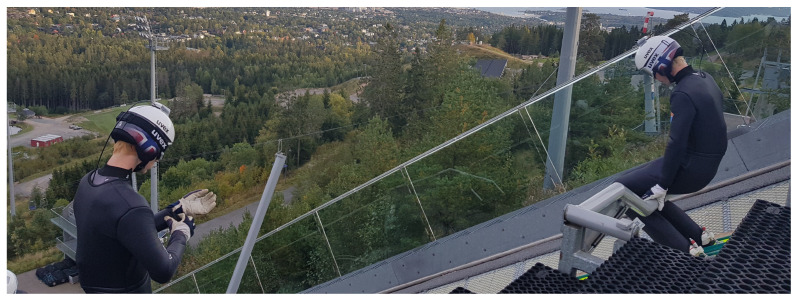
Ski jumpers with the dGNSS antennae mounted on their helmets and the receivers in backpacks that were carried under the ski jumping suit.

Given the applicability, resources and effort needed for video and force measurements, these are often limited to the take-off and early flight phase. Therefore, in recent years, wearable sensors that allow coverage of the entire process from start of the in-run to the landing have been used. Most commonly, inertial measurement units (IMUs) have been used to measure both kinematics and kinetics with promising results [4,26,27,28,29,30,31]. The shortcoming with IMU measures is that accuracy can be questionable when estimating position integrated from acceleration measures [8]. In addition, interpretation of the position data in a global coordinate system is difficult without support from an additional technology [32]. A method that is better suited to providing global position accuracy is differential global navigation satellite system (dGNSS). While this has only rarely been used in ski jumping [33], dGNSS has been extensively used and validated in alpine skiing [34,35,36,37,38]. The use of a dGNSS unit allows measurement of the flight path with ± 0.05 m position accuracy [39]. Once the ambiguities of the differential carrier phase solution are addressed [39], position accuracy also allows the derivation of velocity, the total braking force during the in-run and the aerodynamic forces during the flight phase, by calculation of these as time-position derivatives. Using this system, point mass kinematics and kinetics can be described for the entire period from the start of the in-run to the landing with a single, wearable system. GNSS signal reception through the GNSS antenna requires a direct line of sight to the satellites and is therefore constrained to being mounted on the back or head of the ski jumper, as shown in Figure 2. The antenna mounting point can be considered a reasonable representation of the athlete as a point mass in the stable flight phase and in-run, but not in the take-off, where athletes change body extension and orientation within a short period of time. Thus, an insufficiently accurate representation of the centre of mass position (CoM) occurs during this phase.

The stable flight phase is a commonly used term in ski jumping literature, referring to the aerial phase where the ski jumper has reached a fairly constant flight position, and which lasts until the landing starts. It is worth noting that the term ‘stable’ is misleading since the state of stability is irrelevant. From a physics point of view, this phase should be described as ‘isometric-static’; i.e., acceleration of some key parameter equals zero (some forces seem in balance), and so is the velocity of these parameters. The use of the term ‘stable’ implies that other phases are ‘unstable’, meaning that some perturbation would lead to disastrous changes of position. In the same sense, the term ‘flight’ is not entirely appropriate; physically, ski-jumping resembles more a state between ‘falling’ and ‘gliding’. However, since the results from this paper will be compared to studies referring to this as the stable flight phase, the word ’flight phase’ will be used to avoid misunderstanding.

A better understanding of technique in ski jumping is needed to enhance performance. The above description of the ski jumping methodologies shows that one method alone cannot capture enough relevant data to describe performance and the underlying parameters. We therefore proposed a combination of two methods that have to date not been extensively tested in ski jumping research, which will allow measurement of the kinematics and kinetics from start of the in-run to the landing, including the take-off and early flight phase. A dGNSS is used to describe the kinematics and kinetics from start of the in-run to the landing, while a PosEst system is used to describe kinematics and kinetics of the take-off and early flight phase using a body segment model. Accordingly, the aim of the study is twofold: firstly, we addressed the agreement between these two methods in a region where both are assumed to be accurate. Secondly, we illustrated the strength a combination of these two methods can provide in performance analysis. In Part I, the two methods were applied to compare measured trajectories and estimated velocities and accelerations in 16 ski jumps. The data sets were collected simultaneously, time synchronized and the systems agreement was assessed during the early flight phase, where the dGNSS is assumed to be valid. Part II served as a proof of concept of the sensor fusion application by showcasing the type of performance analysis that can be conducted with the systems. For that purpose two jumps, from the same ski jumper and with comparable wind conditions, were chosen. The trajectories, their derived variables and performance outcomes were evaluated against literature data.

## 2. Materials and Methods

The data collection was carried out in Midstubakken (Hill size 106 m) in Oslo, Norway. Two juniors and three Continental Cup ski jumpers voluntarily participated in this study (all males, age: 21.8 ± 1.8 year, height: 1.76 ± 0.05 m). The study was conducted in accordance with the Declaration of Helsinki, approved by the Norwegian Centre for Research Data and the ethical committee of the Norwegian School of Sport Sciences. The study was conducted over a span of two days. Three ski jumpers were tested the first day and two the next day. A total of sixteen jumps were used for the analysis. The kinematics of the jumps were captured with a camera covering the take-off phase and by a dGNSS. Wind and jump length were measured using the standard International Ski Federation (FIS) competition measurement methodology, where the wind is measured at five points from the in-run to the k-point and the different measurements are weighted to improve fairness in the sport [40].

### 2.1. Video Based PosEst Method

The PosEst system estimated the *x* and *y* coordinates of 16 body parts (i.e., head top, upper neck, shoulders, elbows, wrists, upper chest, right/mid/left pelvis, knees, and ankles) in the video frames in a frame-by-frame manner using a state-of-the-art convolutional neural network (ConvNet) for high-precision pose estimation. For further technical details about ConvNet, the reader is referred to [41]. To perform pose estimations of the ski jumpers, the PosEst was trained, validated and tested on 5064 randomly selected video frames (i.e., 3686 (73%) for training, 365 (7%) for validation, and 1013 (20%) for testing) from an internal database of jumps recorded between January 2014 and June 2019. All 5064 video frames were manually annotated by three experts to define the positions of the body parts, and the PosEst was fine-tuned against these. The video data was captured with one camera (Camera: Blackmagic Pocket Cinema Camera, Lens: Sigma 18–35 mm). The camera was placed 7 m into the flight (relative to the in-run edge) and at a 28 m distance away from the trajectory. The camera view is shown in Figure 1.

The camera filmed in 4K (4096 × 2016 pixels) at 60 Hz with an electronic shutter speed of 1000 s^−1^. The camera was horizontally levelled and slightly angled on the trajectory to capture as much as possible of the flight and the take-off (6.5 m prior to take-off to 22 m after). The image space was calibrated using the ski jumpers segment lengths, measured with a measuring tape prior to testing. The image calibration was conducted by first rotating the image plane in such way that the average segment length of the leg, thigh and arm of the ski jumpers in the 16 jumps remained constant during the flight. Second, calibration was achieved from the image space in pixels to the object space in meters using the athletes’ segment lengths.

The PosEst system required the whole of the ski jumper to be visible in the picture. Each video was trimmed to start at the first, and end at the last, picture where the complete ski jumper was in the picture. A standardised function was created to automatically crop a 500×400 pixel frame around the ski jumper and move frame by frame with the ski jumper. Both the size of the frame and the automatic cropping function were defined manually in the post-processing and the same frame and function were used for all jumps. The camera view, together with examples of how the pictures were cropped and tracked in the in-run, take-off and flight are shown in Figure 1. The area analysed for all jumpers was from −5 to 20 m relative to the jump edge. This experiment had the benefit of not having much human interference in the camera view during the experiment. However, this will not always be the case when filming and it can also be difficult to control. Even if the system is trained to recognise the ski jumper, the appearance of other people or objects in the background can compromise the intentional track. By cropping the picture before tracking one can ensure that the ski jumper is the only human in the tracked picture, hence one will have a less chance of losing data. As seen in Figure 1, another person is present in the lower part of the camera view but is not visible in the cropped pictures and does not interfere in any way. Even if the PosEst located the ski jumper as a 3D object in the picture, the landmarks on the side furthest away had a higher uncertainty. Thus, this study only used the landmarks on the right side for a 2D analysis. This was seen as sufficient, as the movement of the ski jumper primarily occurred in the sagittal plane [23]. CoM was calculated from the PosEst-based joint centre landmarks, the anthropocentric parameters were according to Zatsiorsky with De Leva [42] adjustments, and they were adjusted for the mass of helmet, boots and skis [43].

### 2.2. dGNSS Based Method

The athletes’ head trajectories were captured using a dGNSS with a receiver mounted on the back and an antenna on the helmet, as shown in Figure 2, and a base station positioned in the adjunct to the outrun of the hill. This setup allowed for a short baseline dGNSS calculation. The GNSS antenna (G5Ant-2AT1, Antcom, Torrance, CA, USA) was attached to the helmet of the athlete and connected to the GNSS receiver (Alpha-G3T, Javad, California, USA). The GNSS base station was mounted on a tripod and equipped with an antenna (GrAnt-G3T, Javad, San Jose, CA, USA) and a receiver (Alpha-G3T, Javad, San Jose, CA, USA). The GNSS on the athlete and base station logged 50Hz, GPS/GLONASS dual frequency (L1/L2) GNSS data starting from 30 min prior to the first jump. Raw GNSS data was downloaded from the GNSS receivers and dGNSS solutions were calculated in post-processing.

The GNSS base station was accurately positioned in an absolute global frame WGS 84 (Universal Transverse Mercator zone 32, Northern Hemisphere) by post-processing of its dGNSS position with data from the DPOS base station, Opera, Oslo, Norway provided by the Norwegian Mapping authorities (Hønefoss, Norway) and the geodetic post-processing software Justin (Javad, San Jose, CA, USA). The GNSS antenna position was calculated by use of the geodetic post-processing software Justin in double difference carrier phase mode. This was done to achieve accurate antenna positions from frequencies L1 or L1 and L2 and GPS and GLONASS satellite systems [39]. When the post-processing failed to fix ambiguities (integer ambiguities), float ambiguities (real number ambiguities) were calculated. The processed position data was transformed from the WGS 84 coordinate system to a local coordinate system with the origin at the in-run edge and *x* along the longitudinal axis of the jump and *y* along the gravity vector using a Helmert transformation. This local coordinate system was used for the dGNSS and PosEst analysis.

### 2.3. Parameter Definition and Calculation

**Figure 3:**
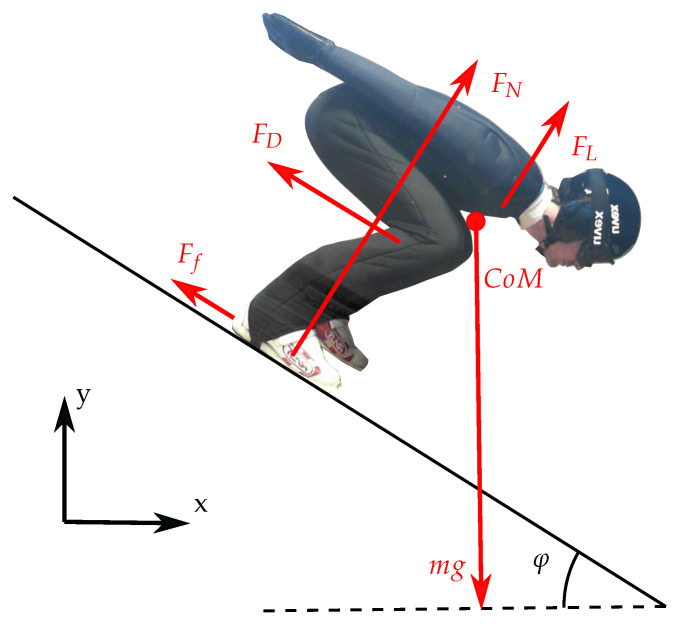
Gravitational (mg), normal ($F_{N}$), friction ($F_{f}$), drag ($F_{D}$) and lift ($F_{L}$) forces acting on a ski jumper in an in-run, together with the coordinate system and angle of the hill (φ).

Performance parameters were calculated from raw position data for both measurement systems (PosEst and dGNSS). Filtering and parameter calculations were conducted in the same manner for both systems. The parameter definitions and calculations are addressed in this section for the different phases of a ski jump. The forces acting on the ski jumper in the in-run are illustrated in Figure 3.

During the in-run, the normal force will have a substantial increase when the ski jumper is entering the curved section due to the centrifugal force and the normal force can be formulated as

(1) $$F_{N}=m\left(g\cos\left(\varphi \right)+\frac{v^{2}}{r}\right),$$

where *m* is the skier’s mass, *g* the gravitational acceleration, *v* the relative velocity and *r* the radius of the curve. The aerodynamic forces are defined as acting parallel (drag) and perpendicular (lift) to the direction of relative motion [44], and are defined as

(2) $$F_{D}=\frac{1}{2}\rho v^{2}C_{D}A$$

and

(3) $$F_{L}=\frac{1}{2}\rho v^{2}C_{L}A,$$

where ρ is the air density, *v* the relative velocity, *A* the frontal area of the object and $C_{D}$ and $C_{L}$ the drag and lift coefficients. The friction force will also act parallel to the motion and is defined as

(4) $$F_{f}=\mu F_{N},$$

where μ is the coefficient of friction. The total force acting parallel to the direction of motion, i.e., braking the ski jumper, can be summed up as the braking force where $F_{b}=F_{D}+F_{f}$. In the same way, the total force acting perpendicular to the motion can be summed up as $F_{p}=F_{N}+F_{L}$. Due to the nature of the ski jump, the direction of the ski jumper may change rapidly at particular moments. The angle between the direction of motion and the global coordinate system will be

(5) $$\varphi =\arctan\left(\frac{v_{y}}{v_{x}}\right),$$

where φ<0. Hence, the local coordinate system of the ski jumper can be found by rotating the coordinate system as follows

(6) $$\left[\begin{array}{c} x^{\prime }\\ y^{\prime } \end{array}\right]=\left[\begin{array}{cc} \cos(\varphi ) & \sin(\varphi )\\ -\sin(\varphi ) & \cos(\varphi ) \end{array}\right]\left[\begin{array}{c} x\\ y \end{array}\right]$$

In this local coordinate system, $F_{x^{\prime }}=F_{b}$ and $F_{y^{\prime }}=F_{p}$. Thus, the braking and perpendicular forces acting on the ski jumper can be found from

(7) $$F_{b}=m\ddot{x}\cos\left(\varphi \right)+m\left(\ddot{y}+g\right)\sin\left(\varphi \right)$$

and

(8) $$F_{p}=-m\ddot{x}\sin\left(\varphi \right)+m\left(\ddot{y}+g\right)\cos\left(\varphi \right).$$

**Figure 4:**
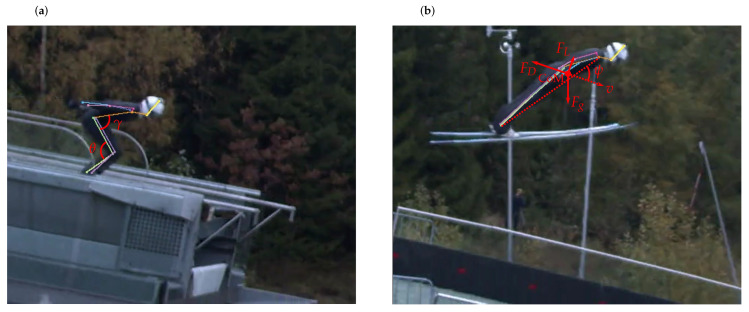
Definition of parameters calculated from the PosEst data: (**a**) shows the knee angle (θ) and the hip angle (γ). (**b**) the centre of mass (CoM), gravitational force ($F_{g}$), drag force ($F_{D}$), lift force ($F_{L}$), body angle of attack (ψ) and the resultant speed of the ski jumper (*v*).

The dGNSS cannot estimate $F_{f}$, $F_{D}$, $F_{N}$ and $F_{L}$ separately but only as the sum of the forces acting parallel and perpendicular to the ski jumper, hence $F_{b}$ and $F_{p}$ are analysed in the in-run. The in-run in ski jumping has a low friction and a high speed, thus the main component of $F_{b}$ is assumed to be the drag force [6]. The main component of $F_{p}$ is assumed to be $F_{N}$, both because this will increase through the curved section and because $F_{L}$ is assumed to be small [45]. As soon as the ski jumper is air-borne both ski friction and normal force vanish and $F_{b}=F_{D}$ and $F_{p}=F_{L}$. Hence drag and lift are analysed for the flight phase. The lift-to-drag ($F_{L}$/$F_{D}$) ratio is also presented as this is seen as an important parameter to describe the quality of the flight phase [46,47]. The knee angle (θ), hip angle (γ) and body angle of attack (ψ), defined in Figure 4, together with their angular velocities, were derived from the PosEst systems landmarks of ankle, knee, hip and shoulder.

The body angle of attack was defined as the angle between the vector from ankle to shoulder and the velocity vector. In recent literature, this parameter is considered equal to the sum of the angle between the skis and the body, and the ski angle of attack (ψ=β+α) [20,21,22]. For simplicity, a summary of the parameters and the phases in which the parameters and methods were deployed is presented in Table 1.

The raw positions data of the antenna/head position from both systems were smoothed with a fourth-order digital zero phase Butterworth filter with a cut-off frequency of 5 Hz [48]. The same filter was applied both before derivation from position to speed and speed to acceleration.

### 2.4. Part I: Comparison of PosEst and dGNSS Method

The dGNSS and the PosEst systems were compared for trajectory, speed and acceleration, with the top head point as the reference, as this was the position of the dGNSS antenna. The instantaneous position of the dGNSS antenna was outputted from the geodetic dGNSS post-processing, and for the video method the top head position was annotated through the PosEst system. The local coordinate system from the dGNSS measurement was applied for both systems with the origin at the edge of the in-run, with *x* being the longitudinal axis and y the vertical axis. The axis cross-track was neglected in the description of the results. For comparison of the methods, both the average data of all 16 jumps and paired differences are presented.

For statistical analysis, t-tests for the time series of the paired difference of each jump were performed using statistical parametric mapping (SPM) with the open-source software package SPM-1D (1-dimensional SPM, www.spm1d.org (accessed on 8 March 2021); ©T.C. Pataky). In this method, a t-test is computed for every time point and Random Field Theory [49] is used to compute a threshold test-value (based on the significance level and smoothness of data) and an overall *p*-value for supra-threshold clusters, instead of calculating a *p*-value for every point. SPM is a relatively new statistical technique in biomechanics, which allows presentation of the time series and statistics graphically, easing the interpretation of the data [50]. The statistical analyses were performed in Matlab R2019b and the level of significance set to α = 0.05. In addition, mean absolute error (MAE) between the paired difference of the jumps and zero difference between the methods (i.e., the assumption of the data being identical as they are from the same source) were calculated for each stretch of 5 m along the trajectory to assess how the error between the methods developed throughout the area under analysis.

### 2.5. Part II: Case Study

As an illustration of this sensor fusion used to assess performance, two jumps by the same athlete and during the same training session were compared in a case study. The jumps were chosen on the basis of similarities in wind conditions, as wind is an important performance-determining parameter in ski jumping [40]. By using the same athlete for both jumps, the effect of body weight can be neglected, which is, together with potential equipment variations between athletes, also an important performance variable in ski jumping [19,51].

The analyses are presented in chronological order, following the two jumps through the in-run, take-off and flight phase to highlight the differences and similarities that lead to a performance difference. The data from dGNSS were used to analyse the in-run and flight phase. During the take-off section the CoM data calculated from the PosEst system was used since the head position (dGNSS) is assumed to be an inaccurate representation of CoM in the section where the ski jumper moves from the in-run position to the constant flight position. Table 1 shows an overview over the performance parameters measured in the different phases and the methods that were used for the analysis. No statistical analysis was performed as this part only consisted of two jumps.

## 3. Results and Discussion

### 3.1. Part I: Comparison of PosEst and dGNSS Data

**Figure 5:**
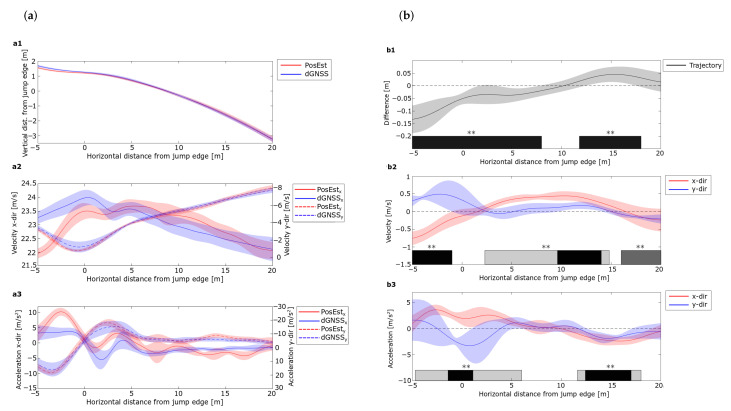
Comparison of data of the PosEst system and the dGNSS. (**a**) show the average trajectory (**a1**), vertical and horizontal velocity (**a2**) and acceleration (**a3**). The shaded error bands indicate the standard deviation with *n* = 16. PosEst data are indicated in red and dGNSS in blue. (**b**) shows the average paired difference of the same variables between the systems. The shaded error bands indicate the standard deviation with *n* = 16 and the dashed line zero difference. The paired differences in the x-direction are indicated in red and in the y-direction in blue. The SPM statistical information is represented by the horizontal bars for the paired difference. The black bar represents the statistical difference between the paired difference and zero in (**b1**). In (**b2**,**b3**), the light gray color represents statistical difference in the x-direction, dark gray in the y-direction and black in both directions, where ** denotes *p* < 0.001.

Figure 5a shows the average trajectory, vertical and horizontal components of velocity and acceleration, together with the SPM analysis. The average paired difference between each of the 16 jumps measured with the PosEst and dGNSS, together with the SPM analysis are presented in Figure 5b. The SPM t-test plots for all variables can be found in Appendix A. Mean absolute error was calculated for phases of 5 m for a better understanding of the magnitude of the difference, presented in Table 2.

A significant difference between the methods was observed for all variables from −5–0 m, with an MAE of around twice the accuracy of the dGNSS (± 0.05 m). Regions of significant trajectory difference from zero between the two methods were also observed after 0 m; however, MAE was smaller than 0.05 m, i.e., within the expected accuracy for the dGNSS [39]. As for the trajectory, the MAEs of both the velocity and acceleration components were large for the first phase and seen to decrease. Regions where the paired difference deviated from zero in the vertical direction were observed in the flight phase, with an MAE of ± 0.15 m s^−1^ and ± 0.97 m s^−2^ as a maximum for the velocity and acceleration respectively. The largest difference was observed in the horizontal direction, with an MAE of ± 0.41 m s^−1^ and ± 1.56 m s^−2^ as a maximum for the region 5–20 m. Altogether, the uncertainty in the measurements was high the first 5 m and deviated from the edge of the in-run. All measurements showed good agreement from 5 m after the in-run. The consistency and agreement 5m indicates that these methods are reliable for use in such analyses.

### 3.2. Part II: Case Study

#### 3.2.1. External Data

The two jumps in the case study were chosen on the basis that they were performed by the same athlete, with the same equipment and similar wind conditions. Gate, wind conditions and jump distances are presented in Table 3.

All wind measurements are shown, to highlight the similar wind conditions during the complete jump. With the given wind compensation, the difference in points between these two jumps would have been 0.9 points (10.5 points initially, minus 9.6 points in gate compensation) in competition. As the jumps were performed by the same athlete during one training session, the body mass and equipment of the ski jumper did not influence the comparison. The difference in in-run speed expected when switching from Gate 15 to 12 on this hill was simulated to be 0.33 m s^−1^. For more information about the simulation, the reader is referred to [6].

#### 3.2.2. In-Run

**Figure 6:**
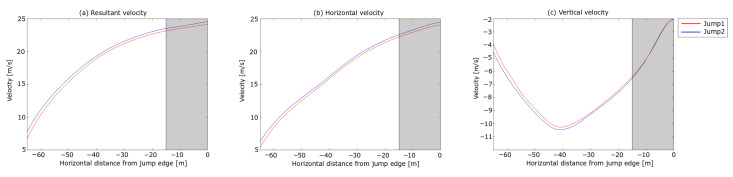
Velocity components of the in-run phase for the two jumps in the case analysis. (**a**) shows the resultant velocity *v*, (**b**) the horizontal velocity $v_{x}$ and (**c**) the vertical velocity $v_{y}$. The horizontal length from the in-run edge is shown on the x-axis and velocity is shown on the y-axis.

The analysis started with dGNSS measurements of the ski jumper leaving the start gate to the take-off (i.e., the in-run phase). The trajectories of the jumps in the in-run are not presented, since no spatial difference was observed between the two jumps in this phase. The last 15 m is greyed out in the plots to symbolise the take-off phase , i.e., the region where the dGNSS outcome cannot be assumed a valid measure for the CoM trajectory. The resultant, horizontal and vertical velocities from the dGNSS are shown in Figure 6.

An instant velocity difference of 1 m s^−1^ was observed, with the difference in the velocity components being 0.86 m s^−1^ and 0.60 m s^−1^ in $v_{x}$ and $v_{y}$, respectively. During the in-run, the difference in $v_{y}$ diminished to zero and $v_{x}$ to approximately half, and thus the difference in *v* was 0.43 m s^−1^ approaching the edge of the in-run. About 0.3 m s^−1^ of the speed difference was expected due to the lower start gate in Jump 2 [6], but 0.1 m s^−1^ ( 0.36 km h^−1^, equivalent to the effect of 1 gate) cannot be explained by the gate difference. In other words, Jump 1 showed a 0.1 m s^−1^ better performance regarding in-run speed. This is a substantial difference, since a difference in in-run speed at take-off of ∼ 0.3 m s^−1^ can increase the jump distance by 3.8–10.1 m approximately, depending on hill size and wind conditions [51,52].

**Figure 7:**
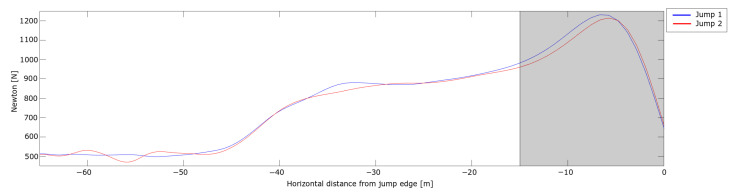
Perpendicular force action on the ski jumper during the in-run. The horizontal length from the in-run edge is shown on the x-axis and velocity is shown on the y-axis.

The measured $F_{p}$ from the dGNSS is shown in Figure 7.

The measured force curves were within the range of recent wind tunnel data [45]. In both jumps, the $F_{p}$ showed a steady increase through the curved section of the in-run and was observed to increasing more rapidly from − 15 m. This force enhancement was due to the athlete’s head no longer following the in-run trajectory, i.e., the take-off action had commenced, around 15 m (around 0.6 s) before the in-run edge. This is almost twice the length and time that the current literature has stated for the take-off [1,2,7]. This may be because of the discrepancy between dynamics and resulting kinematics; the noticeable onset of motion is delayed compared to the onset of forces driving this motion. Thus, a ski jumper may start the take-off action earlier than has been recently assumed.

**Figure 8:**
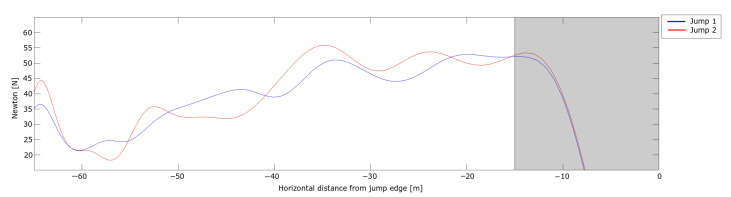
Braking force acting on the ski jumper during the in-run phase measured with the dGNSS. The horizontal length from the in-run edge is shown on the x-axis and velocity is shown on the y-axis.

The total force parallel to the direction of motion, i.e., the braking force is shown in Figure 8.

By neglecting ski friction, one can use Equation (2) to estimate the drag area ($C_{D}A$ value) of the in-run position. By assuming an air density of ρ = 1.225 kg/m${}^{3}$ ( 101.325 kPa at 15 ${}^{\circ }$C), the $C_{D}A$ values of both jumps decreased from around 0.25 to 0.15 m${}^{2}$ throughout the in-run. Here, friction is neglected, thus the actual $C_{D}A$ is expected to be somewhat lower. Nevertheless, this is within the range that Elfmark and Ettema [6] measured for ski jumpers’ in-run positions in a wind tunnel. Hence, the measurements of both the normal force and the braking force were within the range of what has been measured in separate investigations performed in wind tunnels.

The difference in in-run speed is small in the first 25 m of the in-run (Figure 6a). A small velocity difference is expected when starting from different gates as the force producing velocity (gravitation) is constant and the main force resisting (or reducing speed) is drag, which increases with speed squared and hence is small when speed is low at the beginning of the in-run. The estimated forces in the first part of the in-run are quite similar. Even if the speed in Jump 2 is lower, the braking force after entering the curved section of the in-run is higher, which could explain the part of the speed difference that cannot be explained by the different start gates. The grayed area will not be discussed in this section as the head position cannot be assumed to be a valid representation of the CoM during the take-off phase but will be addressed with the PosEst method in the following section.

#### 3.2.3. Take-Off and Early Flight Phase

**Figure 9:**
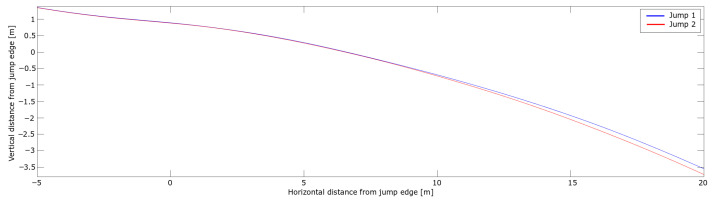
CoM trajectory during the take-off and early flight phase for the two jumps in the case analysis. The horizontal and vertical distance from the in-run edge are shown on the x-axis and y-axis, respectively.

The PosEst system was used for the analysis of take-off and the early flight phase. The trajectories of the CoM of the two jumps for that section are shown in Figure 9.

**Figure 10:**
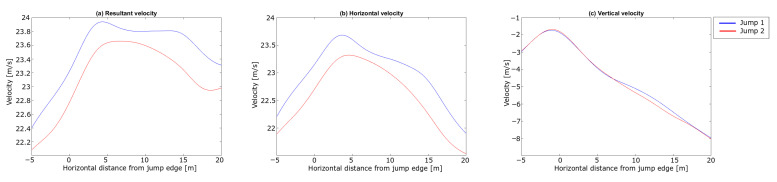
Velocity components of the CoM in the take-off and early flight phase for the jumps in the case study. (**a**) shows the resultant velocity *v*, (**b**) the horizontal velocity $v_{x}$ and (**c**) the vertical velocity $v_{y}$. The horizontal length from the in-run edge is shown on the x-axis and velocity is shown on the y-axis. A negative sign on the x-axis indicates the area before take-off.

A difference in vertical position between the jumps started to emerge around 10 m after the in-run edge, increasing to ∼ 0.17 m. The resultant, horizontal and vertical velocities of the CoM are shown in Figure 10.

**Figure 11:**
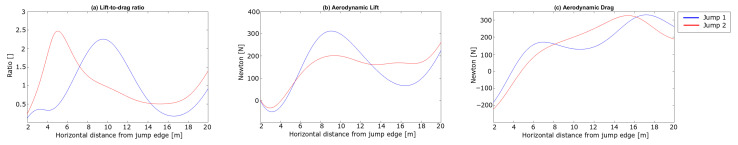
External forces acting on the ski jumper during the early flight phase for the jumps in the case study. (**a**) shows the lift-to-drag ratio ($F_{L}/F_{D}$), (**b**) the aerodynamic lift ($F_{L}$) and (**c**) the aerodynamic drag ($F_{D}$). The horizontal length from the in-run edge is shown on the x-axis. On the vertical axis the unit less ratio is shown in (**a**) and the force [N] in (**b**,**c**).

Similar velocity trends were observed to those seen by both Arndt et al. and Virmavirta et al. [17,22]. The initial speed difference from the in-run is withheld during this phase. Virmavirta et al. [22] found a correlation between the horizontal speed and the jump length. A similar relationship was found in our comparison. Figure 11 shows the lift-to-drag ratio, the aerodynamic lift and the aerodynamic drag during the early flight phase. The forces are shown from + 2 m after the in-run edge to ensure that the skis have left the in-run.

The negative drag at onset of the flight phase (i.e., propulsion rather than resistance) cannot be explained. During take-off, a small component of the push-off force will appear as negative ’drag’ according to Equation (7). This was indeed the case for the signal before + 2 m (not shown). Thus the brief continuation of the negative value may be an outcome of inaccuracy of the PosEst measure. Due to rapid changes in $F_{L}$ and $F_{D}$ in this early flight phase the $F_{L}/F_{D}$ ratio was unstable but increased during the last 5 m (15–20 m). The ratios of $F_{L}/F_{D}$ for the jumps are in the range of what has been previously reported by Schmölzer and Müller [19,20].

**Figure 12:**
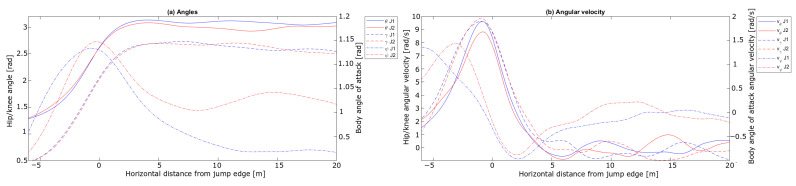
Knee angle (θ), hip angle (γ) and body angle of attack (ψ) measured during the take-off phase for the jumps in the case analysis. (**a**) shows the angles and (**b**) the respective angular velocity. The horizontal length from the in-run edge is shown on the x-axis, angles in rad on the vertical axis in (**a**) and angular velocity in rad s^−1^ in (**b**).

Figure 12 shows the calculated angles and the angular velocity for the two jumps.

For the knee abduction, in Figure 12a, Jump 1 had a steeper increase. The knee angle (θ) was also kept at a larger angle in Jump 1 during the early flight phase. The evolution of the hip angle (γ) for the two jumps seems to be similar. The body angle of attack (ψ) was introduced for the early flight phase in accordance with earlier studies [20,21,22], and also plotted for the take-off phase for comparison (noting its interpretation is different than for the flight phase). Whilst ψ was of a similar value at the in-run edge immediately after take-off, a difference in ψ emerged, which remained constant after ∼ 10 m, where Jump 1 had a lower ψ. Earlier studies have found that a low ψ is beneficial for flight performance [18,21,22,46]. An explanation for why Jump 2 has a higher $F_{L}/F_{D}$ ratio in the last part of this phase may involve the orientation of the skis, which was not included in the PosEst, but influences the aerodynamic forces in a flight [53]. Even if the angular results show that the athlete reaches a stable position in this phase, the $F_{L}/F_{D}$ ratio varies considerably for the complete phase, which will be addressed in Section 3.3. The angular velocities presented in Figure 12b are in the range of earlier lab and field studies [2,54]. Virmavirta et al. [2] found a correlation between the angular velocity of the hip at the release instant and the jump distance. In this case, the angular velocities of the hip showed similar trends between the two jumps, but the jumps differed in peak velocity of the knee.

To summarise, a difference in the CoM trajectory emerged from ∼ 10 m after the in-run edge with Jump 1 being at a higher altitude. The main explanation for this divergence in vertical position is the higher horizontal speed in Jump 1, mainly caused by a higher start gate. The peak force normal to the take-off platform is explained by a higher angular velocity of the knee angle, together with a higher ψ in the take-off, resulting in a more upright position, which might cause a higher take-off force in the vertical direction. Interestingly, the $F_{L}/F_{D}$ ratio varied during the first 20 m of the flight phase, even though the athlete seemed to reach a constant position around 5 to 10 m after the take-off.

#### 3.2.4. Flight Phase

**Figure 13:**
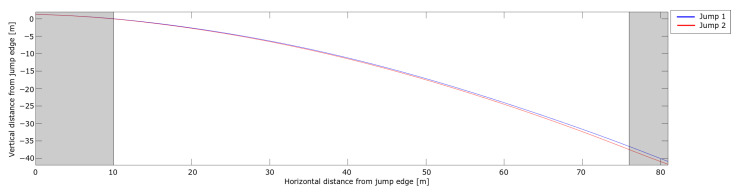
Trajectory from the dGNSS in the flight phase for the two jumps. The gray shaded areas highlight the take-off and landing phases. The horizontal and vertical distances from the in-run edge are shown on the x-axis and y-axis, respectively.

As soon as a ski jumper has reached a constant flight position, the head point will again be a valid representation of the skiers flight path as a point mass. Considering the angular data from 5–10 m after the take-off in Figure 12, the trajectories of the dGNSS in the flight phase of the two jumps are shown in Figure 13, where the early flight phase and landing are gray shaded. The take-off phase has been addressed earlier and the definition of where the landing started will be addressed below.

**Figure 14:**
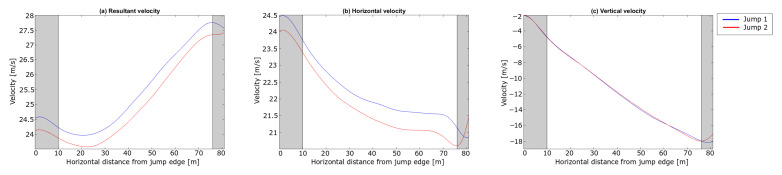
Velocity components in the flight phase for the jumps in the case study. The gray shaded areas highlight the take-off and landing phases: (**a**) shows the resultant velocity *v*, (**b**) the horizontal velocity $v_{x}$ and (**c**) the vertical velocity $v_{y}$. The horizontal length from the in-run edge is shown on the x-axis and velocity is shown on the y-axis.

The difference in the trajectories between the jumps at 20 m was 0.1 m, almost half of the difference measured with the CoM (PosEst) and head (dGNSS) at this point. In Jump 2, the athlete had a larger body angle of attack in the second part of the early flight phase, i.e., a more upright flight position. Hence, the position of the head was higher relative to the CoM for Jump 2, which might explain this difference. The resultant, horizontal and vertical velocities are shown in Figure 14.

**Figure 15:**
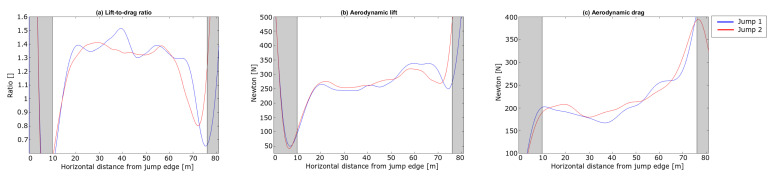
Forces acting on the ski jumper in the flight phase. (**a**) shows the lift-to-drag ratio ($F_{L}/F_{D}$), (**b**) the aerodynamic lift ($F_{L}$) and (**c**) the aerodynamic drag ($F_{D}$). The horizontal length from the in-run edge is shown on the x-axis with a unit less ratio in (**a**) and the forces [N] in (**b**,**c**) on the vertical axis. The gray shaded areas highlight the take-off and landing phases.

The landings of the two jumps were at 80 m and 76 m for Jump 1 and Jump 2, respectively. Here, both the horizontal and vertical velocity start to decrease. The jump distance is measured along the hill profile, so a 4 m horizontal distance corresponds to a total difference of 5 m, as shown in Table 3. Both Figure 13 and Figure 14 show similar trends to another of the rare studies using dGNSS in ski jumping Blumenbach [33]. The resultant speed (Figure 14a) slightly decreased during the early flight phase, as already shown with the PosEst system, and started to increase after 20 m. The increase in resultant speed was caused by the increase in vertical velocity as a result of gravity acting on the ski jumper over time. The increase in the resultant speed was almost linear from 30 m until the ski jumper initiated the landing. The difference in resultant velocity between the jumps remained at 0.4 m/s for most of the flight phase, emphasising the importance of the in-run speed. A sudden drop in horizontal velocity was seen right before the landing, assumed to be the ski jumper opening up his position to prepare for landing. Figure 15 shows the $F_{L}/F_{D}$ ratio, $F_{L}$ and the $F_{D}$ of the flight phase. The $F_{L}/F_{D}$ ratios are relatively constant from around 20 m and 24 m for Jump 1 and Jump 2, respectively, i.e., reaching a constant ratio after ∼ 1 s. This is in accordance with simulations performed by Schmölzer et al. [19]. The ski jumper maintains a ratio of 1.3-1.5, in the constant phase, which corresponds well to earlier simulations and experiments [18,19,20,46,47]. Jump 1 had a longer phase with a constant lift-to-drag ratio and a somewhat higher average for the lift-to-drag ratio. The overall trends of the lift and drag force of the two jumps were similar.

In summary, the differences in speed and technique that were observed in the in-run, take-off and early flight phase led to differences in the flight kinetics during the flight phase. The main effect of the differences in drag and lift forces between the jumps was on the vertical position of the trajectory in the last part of the flight. Due to the squared lift-velocity relationship, the higher speed in Jump 1 led to more lift and consequently a higher vertical position and better performance. Only small differences in drag and lift were detected between the jumps for the main part of the flight, but the velocity differences were systematic. Hence, the ski jumper must have had smaller values of $C_{D}A$ and $C_{L}A$ (see Equations (2) and (3)) in Jump 1, which may have come from the lower body angle of attack (ψ) observed during the early flight phase.

### 3.3. Possibilities and Limitations

This study has introduced the combination of two promising methods (dGNSS and PosEst) for performance analysis in ski jumping, which have not been extensively used in recent ski jumping research. The good agreement between the methods for take-off and the early flight phase, and the analytic possibilities that this sensor fusion can provide, have been presented. The major strength of using dGNSS in ski jumping is the ability to measure the kinematic variables (and their kinetic derivations) from start of the in-run to the landing in a consistent and relatively resource-conservative manner, possibly with better accuracy than, for example, IMU-based estimations of the trajectory [4,26,27,29,30,31]. Since the dGNSS method allows estimation of the external forces, velocity and position instantaneously from start of the in-run to the landing, with good global accuracy, the method can offer a holistic assessment of performance and its underlying factors. Instantaneous tracking of velocity and forces allows not only description of how these parameters develop over time but also an explanation of how these interact and affect performance, albeit with limited validity during the take-off phase. Hence, the dGNSS method alone could help an individual or coach to better understand the reasons behind a given performance. A limitation to this method is that it cannot be used in competition, as the dGNSS has to be mounted on the ski jumper. It is also important to notice that the antenna of this dGNSS was placed on the helmet and is used as a point mass representation of the athlete, which is a reasonable assumption in the in-run and flight phase, but during take-off and early flight phase the head motion substantially deviates from the CoM motion. Mounting of the dGNSS system closer to the pelvic region may partly solve this issue. A complementary method should be used to accurately assess the CoM motion to provide data from what is to date considered to be the most important phase of ski jumping.

The usage of video to analyse the take-off is not new [2,17,18,19,20,21,22], but no research published to date has used a markerless, video-based pose estimation using computer vision. Usage of a PosEst method can reduce the biggest limitation of video analysis, i.e., the time used on data processing. The PosEst was used to assess the take-off and early flight phase from one camera and it was demonstrated that the PosEst method can reconstruct the segment motion of the athlete in the sagittal plane along with CoM position, velocity and estimations of the external forces. This can be done without interfering with the athlete and is thus well suited to competition analysis. The camera-based PosEst method can be applied at any point along the trajectory but may be most important in the take-off phase, which is considered to be the most important phase of ski jumping [1,2,3]. This method also becomes more and more promising as camera technology develops, enabling filming in high resolution with a simultaneous high frame rate. Such a system could also further be improved by an automatic object detection and crop procedure. Covering a larger part of the in-run, jump, flight phase and landing could be achieved by synchronizing several cameras. Calibration of the 3-D space covered by multiple cameras is another important but solvable challenge.

Used together, these two methods cover most of the motion analysis features of ski jumping, except for the ski motion data which are difficult to obtain in the sagittal plane but are nonetheless important in ski jumping [53]. A possible way to capture both the pitch, roll and yaw of the skis could be to place an IMU on each ski and time-synchronise these with the dGNSS and video. IMUs may also be valuable for obtaining kinetic parameters like angular velocities and acceleration of limb segments for the complete ski jump [28]. An interesting feature that could be assessed using the presented sensor fusion is the onset of the constant flight phase. In recent literature, the constant flight phase is assumed to be reached when the ski jumper has a constant flight position [1,20,22]. In this study it was found that the angles and angular velocity, measured at the take-off (Figure 12) started to stabilise between 5 and 10 m, while aerodynamic analysis based on the same data set suggested a constant flight period started at about 20 m. Such discrepancies may provide fruitful insights into the definition and role of the early flight phase and how and when that leads into the constant flight phase.

### 3.4. Summary

This study investigated two methods, not previously used, for performance analysis in ski jumping, which will allow measurement of the kinematics and kinetics from the start of the in-run to the landing. The dGNSS was used to describe the kinematics and kinetics for the in-run and early flight phase to the landing, whilst the PosEst system was used to describe kinematics and kinetics of the take-off and early flight phase, using a body segment model. The study was separated into two parts, where the first part compared the agreement of the systems from 5 m before take-off to 20 m into the flight. Trajectory, velocity and acceleration components were compared between the systems for 16 jumps. Good agreement, within the uncertainty of the dGNSS, was found between the two methods from 5 and 20 m after take-off, indicating that the methods agree well in the flight phase, while PosEst performed better in the analysis of the take-off phase. The second part of the study served as a proof of concept of the sensor fusion application, showcasing the types of performance analysis that can be conducted with this system combination. For that purpose data from two jumps were extracted and used to illustrate how start gate, drag and lift influence the instantaneous velocity vector, altitude above ground and finally jump length. The study showed the complementary nature and validity of the methods and their usability in both research and practice.

## Figures and Tables

**Table 1:** Overview of the performance parameters measured in the different phases of the ski jump and the method used for the analysis. Dist. is the horizontal distance from the jump edge and Tr. the trajectory of the head for the dGNSS and CoM for the PosEst.

Phase	Dist.	Method	Tr.	*v*	$v_{x}$	$v_{y}$	$F_{b}$	$F_{p}$	$F_{D}$	$F_{L}$	$F_{L}/F_{D}$	ψ	θ	γ	$v_{\psi }$	$v_{\theta }$	$v_{\gamma }$
	[m]		[m]	[m s^−1^]			[N]				[]	[rad]			[rad s^−1^]		
in-run	−65–0	dGNSS	X	X	X	X	X	X									
Take-off	−5–20	PosEst	X	X	X	X			X	X	X	X	X	X	X	X	X
Flight	0–90	dGNSS	X	X	X	X			X	X	X						

**Table 2:** Mean absolute error (MAE) for phases of 5 m between the paired difference and zero difference (Figure 5b).

Phase [m]	−5–0	0–5	5–10	10–15	15–20
Tr [m]	0.10	0.04	0.02	0.03	0.03
$v_{x}$ [m s^−1^]	0.49	0.15	0.41	0.34	0.15
$v_{y}$ [m s^−1^]	0.41	0.10	0.08	0.13	0.15
$a_{x}$ [m s^−2^]	2.45	2.19	0.43	1.44	1.56
$a_{y}$ [m s^−2^]	1.44	1.89	0.57	0.97	0.92

**Table 3:** External data for the two jumps chosen for the case study. Distance points and wind points showing the length and wind compensation points in an FIS competition.

	Gate	Distance		Total Wind Score		Wind Measurements at [m s^−1^]				
	[#]	[m]	Points	[m s^−1^]	Points	10 m	38 m	57 m	76 m	95 m
Jump 1	15	96	62	0.40	−3.2	1.63	0.80	0.71	0.52	0.29
Jump 2	12	91	52	0.46	−3.7	1.44	0.94	0.50	0.78	0.92

**Figure A1:**
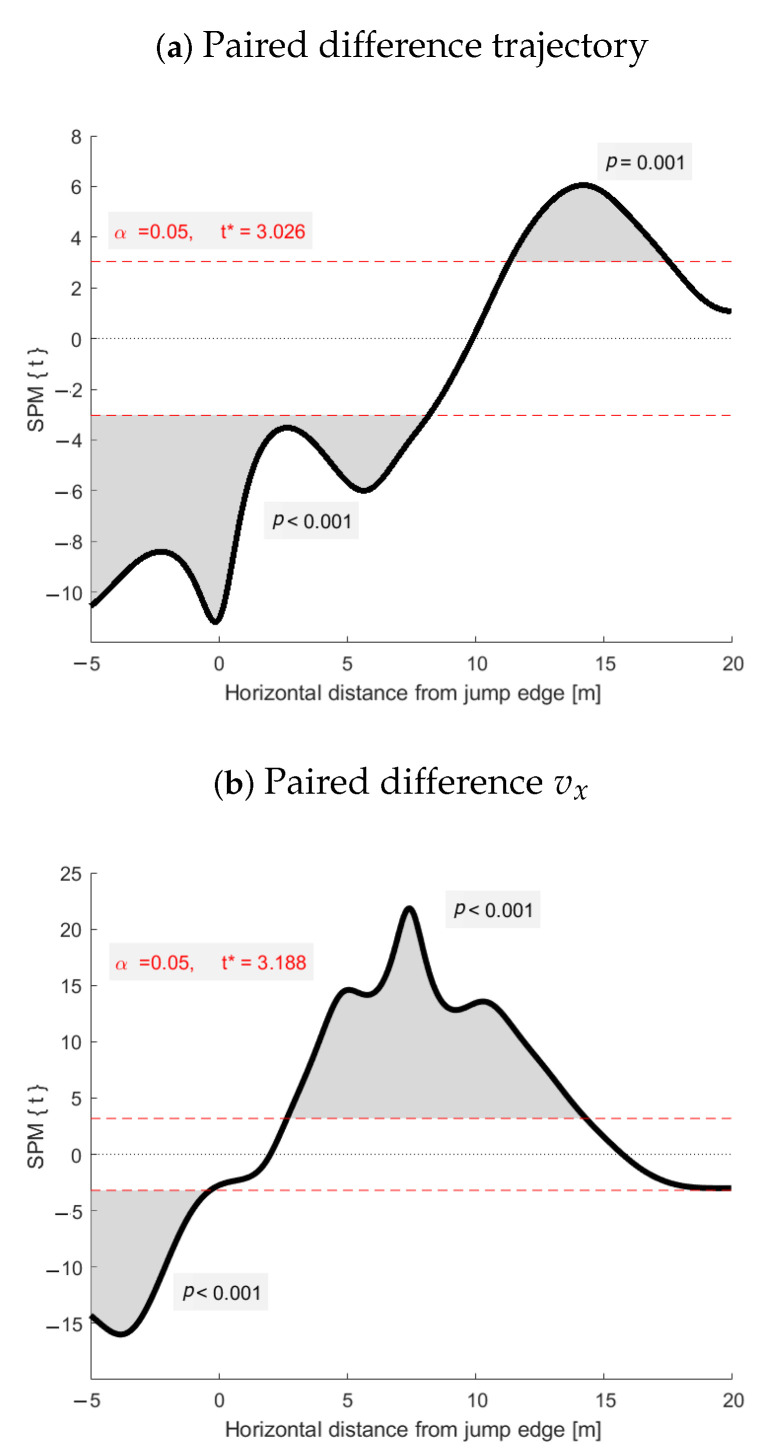

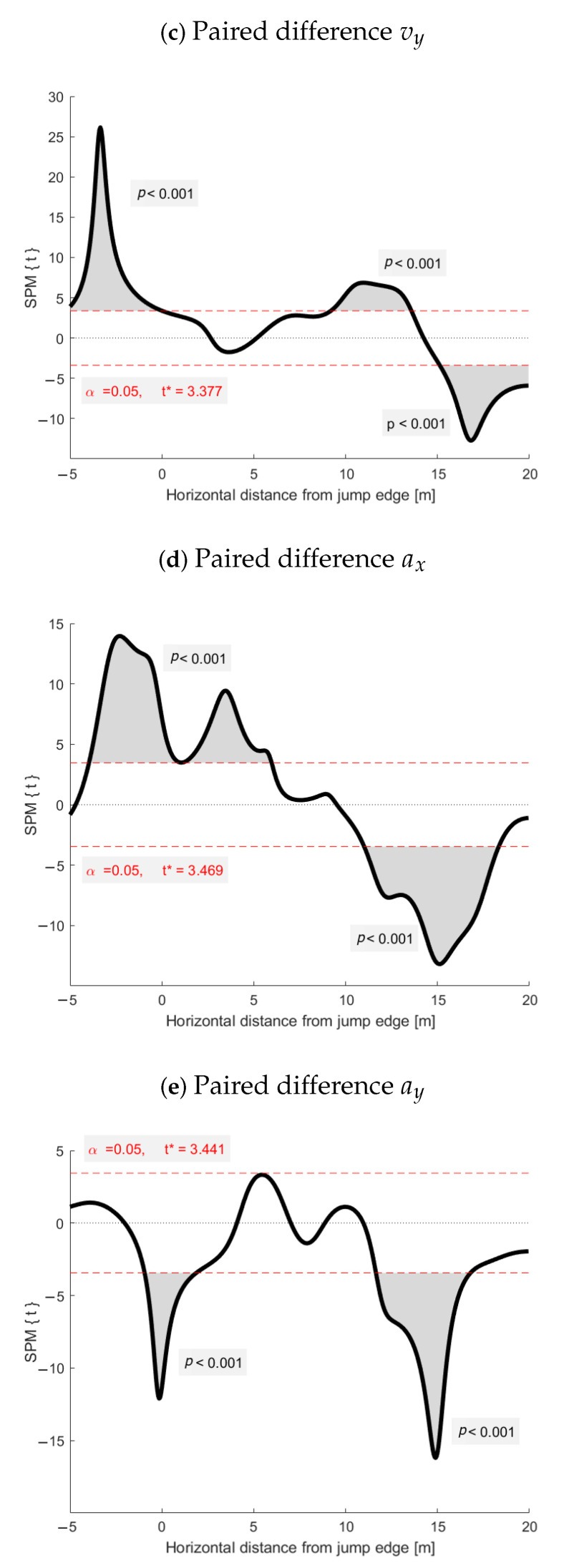
Statistical parametric mapping t-test analysis of the paired difference between the methods compared to zero difference of trajectory (**a**), horizontal and vertical velocity (**b**,**c**) and acceleration (**d**,**e**) (*n* = 16). The red, dashed, horizontal lines indicate the threshold t${}^{*}$ test-values and the values are reported in each plot. Significant supra-threshold clusters are marked with a grey shade, together with their corresponding *p*-values.

## Data Availability

The data presented in this study are available on reasonable request from the corresponding author.

## Abbreviations

The following abbreviations are used in this manuscript:

CoM	Center of Mass
ConvNet	Convolutional Neural Network
dGNSS	differential Global Navigation Satellite System
$F_{b}$	Braking force
$F_{D}$	Drag force
$F_{f}$	Friction force
$F_{L}$	Lift force
$F_{L}$/$F_{D}$-ratio	Lift-to-drag ratio
$F_{N}$	Normal force
$F_{p}$	Perpendicular force
FIS	International Ski Federation
IMU	Inertial Measurement Units
MAE	Mean Absolute Error
PosEst	Markerless Video-based Pose Estimation
SPM	Statistical Parametric Mapping

## Appendix A. Statistical Parametric Mapping Analysis

Figure A1 shows the separate results of the SPM t-test of trajectory and the horizontal and vertical components of velocity and acceleration for the paired difference, compared to zero difference between the methods (presented in Figure 5). The significance level was set to α = 0.05 for all test. The horizontal lines indicate the threshold t${}^{*}$ test-values and the t${}^{*}$ are reported in each plot, together with the *p*-values. The significant supra-threshold clusters are marked in the plots with a grey shade.
